# Advancing learning health care systems with best practice meetings: a mixed-methods study with insights from the Dutch Institute for Clinical Auditing

**DOI:** 10.1093/intqhc/mzag111

**Published:** 2026-08-08

**Authors:** Felix Hers, Lisanne E van Rangelrooij, Janne van den Hurk, Lars van de Sanden, Willemien J van Driel, Grard A P Nieuwenhuijzen, Barend M E Mees, Mira S Staphorst, Mark M J Nielen, Michel W J M Wouters

**Affiliations:** Scientific bureau, Dutch Institute for Clinical Auditing, Leiden, the Netherlands; Department of Vascular Surgery, Maastricht University Medical Center, Maastricht, the Netherlands; Scientific bureau, Dutch Institute for Clinical Auditing, Leiden, the Netherlands; Department of Gynaecology, Centre for Gynecological Oncology Amsterdam, The Netherlands Cancer Institute—Antoni van Leeuwenhoek Hospital, Amsterdam, the Netherlands; Department of Medical Oncology, Amsterdam University Medical Centre, University of Amsterdam, Amsterdam, the Netherlands; Scientific bureau, Dutch Institute for Clinical Auditing, Leiden, the Netherlands; Department of Surgery, Leiden University Medical Center, Leiden, the Netherlands; Scientific bureau, Dutch Institute for Clinical Auditing, Leiden, the Netherlands; Department of Surgery, Catharina Hospital, Eindhoven, the Netherlands; Department of Gynaecology, Centre for Gynecological Oncology Amsterdam, The Netherlands Cancer Institute—Antoni van Leeuwenhoek Hospital, Amsterdam, the Netherlands; Department of Surgery, Catharina Hospital, Eindhoven, the Netherlands; Department of Vascular Surgery, Maastricht University Medical Center, Maastricht, the Netherlands; Scientific bureau, Dutch Institute for Clinical Auditing, Leiden, the Netherlands; Scientific bureau, Dutch Institute for Clinical Auditing, Leiden, the Netherlands; Scientific bureau, Dutch Institute for Clinical Auditing, Leiden, the Netherlands; Department of Biomedical Data Sciences, Leiden University Medical Center, Leiden, the Netherlands; Department of Surgical Oncology, Netherlands Cancer Institute-Antoni van Leeuwenhoek, Amsterdam, the Netherlands

## Abstract

**Background:**

National clinical registries are widely used to benchmark quality indicators and improve care. Advancing registry-based auditing toward learning health care systems (LHCS) requires structural integration of continuous evaluation and improvement cycles. One way to operationalize the check phase of the Plan-Do-Check-Act cycle is through best practice meetings (BPMs), in which health care professionals transparently discuss inter-hospital variation in clinical outcomes and formulate targeted improvement actions. Since 2018, the Dutch Institute for Clinical Auditing (DICA) has organized BPMs across multiple registries. This study provides the first descriptive overview of the structure, participant experiences, and facilitators and barriers to the organization and conduct of BPMs.

**Methods:**

A descriptive mixed-methods study was conducted. Data on session characteristics (e.g. number, topics, format) and follow-up actions were obtained through a survey among coordinators of DICA registries. Participant satisfaction scores from evaluations of BPMs held between 2018 and 2025 were retrospectively extracted, where available. Semi-structured interviews were held with four registry coordinators and one project manager. Interviews were thematically analysed to identify facilitators and barriers to the organization and conduct of BPMs.

**Results:**

In total, 55 BPMs were held across 14 registries. Most were national and multidisciplinary. Frequently discussed topics included length of stay, treatment waiting times, Textbook Outcome, and mortality. Best practices and/or quality improvement actions were formulated for 22 of 55 BPMs (40%). The mean participant satisfaction score was 4.0 on a 5.0-point Likert scale. Five themes were identified through thematic analysis of interviews: preparation, setting and format, learning climate, implementation, and cyclical learning. Facilitators included sharing data in advance, skilled moderation, an open and safe atmosphere, and recurring themes; barriers included an online setting that hampered interaction, concerns regarding data quality, and discussion of too many topics during a single meeting.

**Conclusion:**

BPMs appear to be a feasible and well-accepted instrument to support data-driven quality improvement across hospitals. Implementation of BPMs could support LHCS by operationalizing the learning cycle from benchmarking to shared learning, action planning, and follow-up. Future research should evaluate whether BPMs lead to implementation of improvement actions and measurable changes in clinical outcomes.

## Introduction

In the 20th century, surgical morbidity and mortality conferences (MMC) were introduced to identify opportunities for quality improvement in health care [1]. Currently, registry databases play an important role in monitoring quality of care and targeting areas for improvement [2]. These databases contribute to the emergence of learning health care systems (LHCS), in which routinely collected data fuel continuous cycles of evaluation and improvement [3, 4].

Within an LHCS, registry data are translated into quality indicators (QIs): standardized measures that are compared across hospitals to identify variation and target improvement [5, 6]. Registry-based benchmarking and feedback are associated with measurable improvements, such as reduced postoperative mortality after colorectal cancer surgery [7]. However, providing performance data alone does not always translate into successful quality improvement, and challenges include limited collaboration between centres and inadequate structured follow-up of improvement initiatives [8].

To address these challenges, the Dutch Institute for Clinical Auditing (DICA) initiated best practice meetings (BPMs) in 2018. BPMs are structured sessions in which health care professionals (HCPs) from different medical centres discuss inter-hospital variation in clinical outcome data to identify underlying causes and formulate targeted improvement actions, extending the reflective approach of MMC across hospitals. These sessions emphasize the *check* phase of the Plan-Do-Check-Act (PDCA) cycle, which is commonly used in quality improvement efforts [9], in an interactive format. Similar meetings are often embedded within quality improvement collaboratives and have only been described as part of broader initiatives, rather than as a distinct format [10–12].

The structure, perceived value, and implementation processes of BPMs have not been comprehensively described, and no standardized terminology for such meetings exists, limiting comparability across initiatives [13]. This study aims to address this gap by describing BPM characteristics and participant experience, and by identifying facilitators and barriers to their organization and conduct. These insights could inform other registries seeking to implement similar initiatives.

## Materials and methods

### Study design

This study used a descriptive mixed-methods design, combining a survey across all DICA registries with semi-structured interviews with BPM organizers. The study was reported according to the Standards for Reporting Qualitative Research (SRQR) guideline (Supplementary File 1) [14].

### Context

To promote quality improvement in surgical care, the DICA was established in 2010 and has expanded to 26 nationwide registries encompassing oncological and non-oncological care (Supplementary File 2) [15]. In 2018, BPMs were developed within DICA to translate benchmarked registry data into structured reflection, improvement actions, and follow-up as part of the PDCA cycle. Meeting content was drafted by HCPs affiliated with the registry’s scientific committee, while DICA staff, typically including a project manager, a registry coordinator, and a data analyst, focused on organizing the meeting and the data analysis.

**Figure 1:**
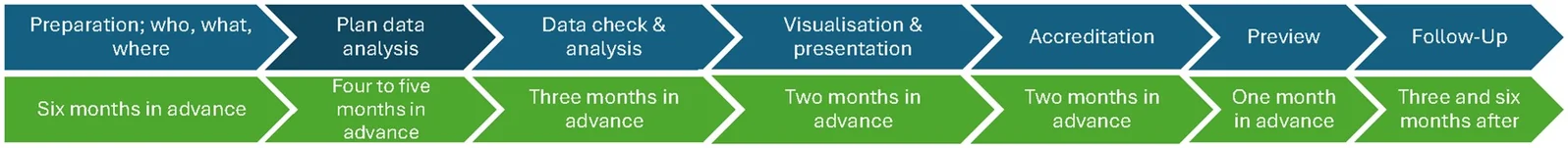
Timeline of the best practice meeting organizational process.

Organizing a BPM involves a structured process: identifying discussion topics, analysing and presenting data, facilitating discussion, formulating best practices or improvement actions, and organizing follow-up meetings (Fig. 1). Topics often concerned benchmarked QIs, such as Textbook Outcome, selected by HCPs based on inter-hospital variation, suboptimal national outcomes, or clinical relevance. When patient numbers for a topic were limited, questionnaires were distributed to HCPs beforehand to complement the audit data (e.g. on hospital-specific protocols or data from the patient file).

### Data sources and inclusion

Data related to all BPMs held between 2018 and 2025 were obtained from three sources: (i) an internal survey sent in December 2025 to the registry coordinator for each of the 26 DICA registries, (ii) routine BPM participant evaluations, where available, and (iii) semi-structured interviews with four registry coordinators and one project manager selected because they had experience in the organization of at least two BPMs.

Registries for which the survey response stated that no BPM had been organized were excluded from the analysis.

### Data collection

#### Survey

Session characteristics were collected using an internal survey with 12 questions (Supplementary File 3), capturing the number of BPMs, meeting format, organizational level, multidisciplinary participation (i.e. attendance of at least two different HCPs: medical specialists, paramedics, nurses and/or other HCPs), discussion topics, and use of follow-up meetings per registry. The attendance rate (attending centres divided by invited centres, if available), participation of patient representatives (in preparation or during the BPM), and formulation of improvement actions (yes/no) were collected per BPM.

#### Participant evaluations

Participant experience was extracted retrospectively from routine BPM evaluations conducted using online surveys and interactive tools (e.g. Mentimeter [16]) (Supplementary File 4). Where available, overall satisfaction scores and other measures, such as intention to attend future BPMs, were collected from the online platform.

#### Interviews

Semi-structured interviews were conducted in Dutch (L.E.v.R.), guided by a topic guide (Supplementary File 5), audio-recorded, and transcribed verbatim. Interviews focused on BPM preparation, participant selection, use of data and interactive tools, moderation, and the formulation, follow-up, and evaluation of improvement actions, and their perceived effects on clinical practice.

### Data analysis

Session characteristics and survey data were analysed descriptively per registry or, if specific data were available, per BPM. Discussion topics, improvement actions, and follow-up activities were reported separately. Satisfaction scores were summarized using means.

Transcripts were analysed using thematic analysis following the method described by Gale *et al.* [17] Two transcripts were coded independently by two researchers (L.E.v.R. and F.H.), after which codes were compared and discussed to establish a shared coding approach. The remaining three transcripts were coded by one researcher (L.E.v.R.). Subsequently, three researchers (L.E.v.R., F.H., and M.M.J.N.) collaboratively organized codes into preliminary themes and subthemes. The remaining organization of codes was carried out by one researcher (L.E.v.R.). Final theme development was discussed within the wider team (L.E.v.R., F.H., and M.M.J.N.). Analysis was inductive, driven by the data rather than a pre-existing theoretical framework. Themes were refined iteratively until a coherent thematic structure was established.

### Ethical considerations

This study used retrospective, aggregated data from routine BPM evaluations and internal surveys. No patient-level data were collected or analysed. According to Dutch regulations, survey-based research and anonymized interviews that do not involve patient data, clinical interventions, or identifiable information do not fall under the Medical Research Involving Human Subjects Act (WMO) [18]. Ethical review was therefore not required.

## Results

### Structure and participation

All 26 registry coordinators from DICA registries responded. Survey results indicated that 12 registries had never organized a BPM and were therefore excluded. In total, 55 BPMs were organized by 14 DICA registries since 2018. The highest number of BPMs was organized by the medication audit (*n* = 10), the head and neck oncology audit (*n* = 7), and the upper GI cancer audit (*n* = 7) (Table 1). The paediatric surgery audit was the first registry to organize an international BPM. BPMs were held at a national level in 13 of the 14 registries (93%), were multidisciplinary in nine (64%), and were most often held face-to-face (Table 1). The attendance rate was available for 17 BPMs. The highest attendance rate was observed for the gynaeco-oncology audit BPMs, with participation from all invited medical centres. Patient representatives contributed to the preparation or attended for 10 of 55 BPMs (18%).

**Table 1:** Best practice meeting structure and topics per DICA registry.

Audit	Number of meetings	Multidisciplinary	Platform	Meeting	BPM discussion topics	Improvement actions	Follow-up meeting
Bariatric surgery	Three	No	Online meetings	Nationwide	One-day admissions, preoperative weight loss, patient characteristics, postoperative bleeding, and Textbook Outcome.	Yes	No
Breast cancer	Two	Yes	Face-to-face and online meetings	Nationwide	Appropriate and efficient use of systemic anticancer medicines, based on outcomes (e.g. survival, time to next treatment) and practice variation; e.g. specific targeted therapies.	Unknown	No
Colorectal cancer	Four	Yes	Face-to-face and online meetings	Nationwide	Appropriate and efficient use of systemic anticancer medicines, based on outcomes (e.g. survival, time to next treatment) and practice variation; e.g. use of immunotherapy shortly before the end of life.	Unknown	No
Gastrointestinal endoscopy and complications	One	No	Face-to-face meeting	Nationwide	Complication rates, Quality indicators (e.g. polyp detection ratios), and the structure of morbidity and mortality meetings.	No	No
Gynaeco-oncology	Three	No	Face-to-face meetings	Nationwide and regional	Cervical and ovarian cancer. Adjuvant therapy, electronic (regional) benchmarking dashboard (Codman), length-of-stay, complications, surgical approach, wait times for surgery and adjuvant treatment, and trends in treatment over time.	Yes	Yes
Head and neck oncology	Seven	Yes	Face-to-face and online meetings	Nationwide	Completion rate of chemotherapy, complicated admissions, PROMs, wait times for adjuvant treatment after surgery, wait times for treatment, and unplanned reintervention.	Yes	Yes
Hepato-Biliary Cancer	Three	Yes	Face-to-face meetings	Nationwide	Application of diagnostic MRI, prehabilitation, preoperative liver function testing, outcomes after ablation, and volumes per hospital.	Yes	No
Hip fracture	One	Yes	Face-to-face meetings	Regional	Length-of-stay, surgical wait times, treatment of osteoporosis, type of treatment per fracture type, and volumes per hospital.	Yes	No
Lung cancer	Five	Yes	Face-to-face and online meetings	Nationwide	Appropriate and efficient use of systemic anticancer medicines, based on outcomes (e.g. survival, time to next treatment) and practice variation in lung cancer; e.g. dosing and maintenance treatment strategies for immune checkpoint inhibitors and chemotherapy.	Unknown	No
Medicine usage	Ten	Yes	Face-to-face and online meetings	Nationwide	Non-disease-specific sessions on efficient and durable usage of medicine based on outcomes (e.g. survival, time to next treatment, or death) and variation in medication usage.	Unknown	No
Paediatric surgery	Five	Yes	Face-to-face meetings	International, nationwide, and regional	Data completeness, general patient characteristics, and specific complications.	No	No
Prostate cancer	One	Yes	Face-to-face meeting	Nationwide	Appropriate and efficient use of systemic anticancer medicines, based on outcomes and practice variation in metastatic prostate cancer; e.g. treatment sequencing in low- and high-volume disease.	Unknown	No
Stroke	Three	No	Online meetings	Nationwide	Data completeness, Door-to-Door time, Door-to-Needle time, initial management at the emergency department, interhospital care, prehospital phase, and regional electronic dashboard usage.	Yes	Yes
Upper gastrointestinal cancer	Seven	No	Face-to-face and online meetings	Nationwide	Length-of-stay, peri-operative complications (e.g. anastomotic leakage), surgical techniques, treatment wait times, and lymph node retrieval	Yes	Yes

Abbreviations: BPM, best practice meeting; MRI, magnetic resonance imaging; PROMs, patient-reported outcome measures.

### Discussion topics, improvement actions, and follow-up

Topics that were frequently discussed during BPMs included procedure-specific complications, length of stay, waiting times for treatment, postoperative bleeding, Textbook Outcome, and mortality (Table 1). Best practices and/or quality improvement actions were defined for 22 of the 55 BPMs (40%). Follow-up meetings to evaluate implementation were scheduled after BPMs for 4 of the 14 registries (29%).

### Participant experience

**Figure 2:**
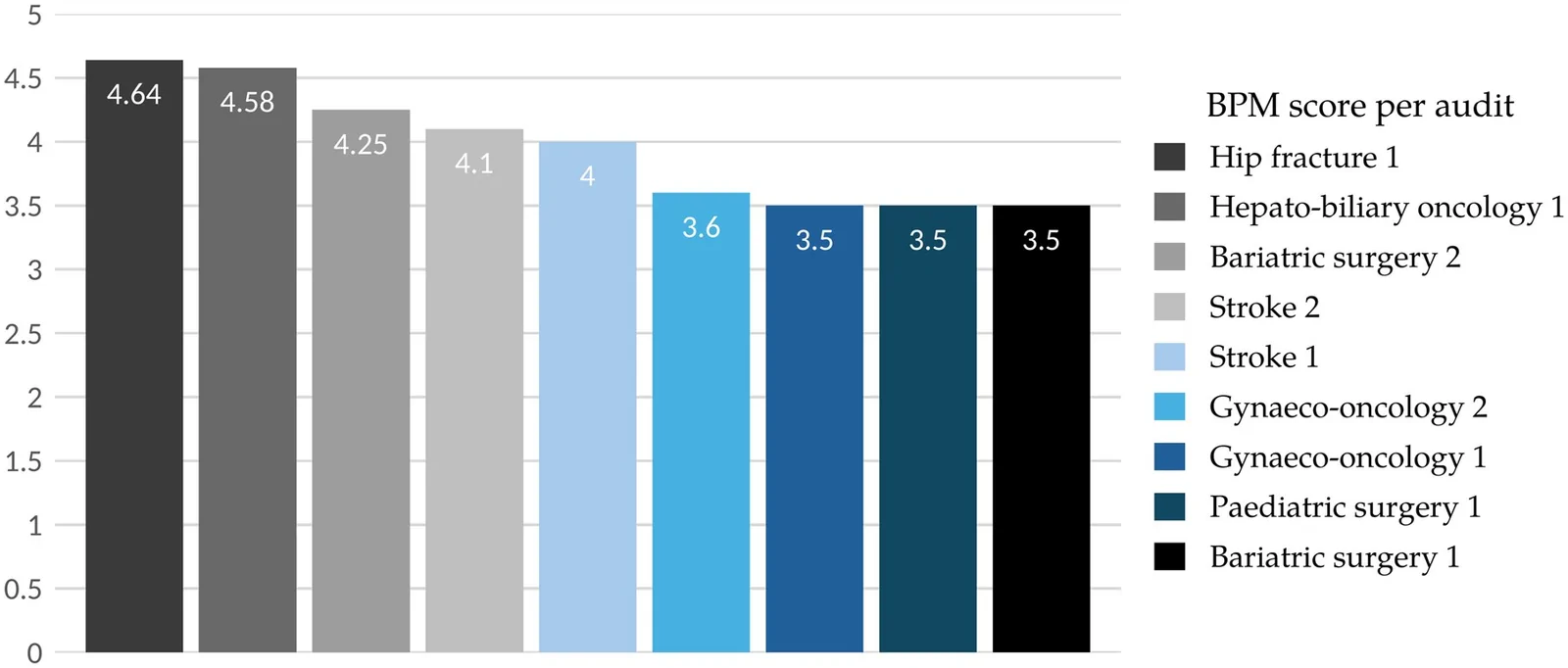
Mean participant satisfaction per best practice meeting (5-point Likert scale: 5 = very satisfied; 4 = satisfied; 3 = neutral; 2 = not satisfied; 1 = not satisfied at all).

Participant feedback on meeting quality was collected using digital surveys or Mentimeter polls for participating HCPs. Digital surveys were used by two registries (14%) and Mentimeter by six registries (43%) that organized BPMs. The participant satisfaction score was registered for 9 of 55 BPMs, with a mean score of 4.0 on a 5.0-point Likert scale. The hip fracture audit and hepato-biliary oncology audit received the highest mean scores (4.64, *n* = 14 and 4.58, *n* = 26, respectively) (Fig. 2). All participants of the hip fracture audit BPM indicated that they would attend a future BPM.

### Facilitators and barriers

Through thematic analysis of participant interviews, multiple facilitators and barriers to the organization and conduct of BPMs were identified, which could be grouped into 25 subthemes and 5 overarching themes (Table 2): preparation, setting and format, learning climate, implementation, and cyclical learning. Illustrative quotes are presented per theme.

**Table 2:** Facilitators and barriers related to the organization and conduct of best practice meetings.

**Theme**	**Subtheme**	Facilitators	**Barriers**
**Preparation**	**Data analysis and dashboard**	An interactive dashboard is suitable for exploring practice variation during preparation An interactive dashboard answers participants’ questions directly and stimulates discussion	Data analysis is highly time-intensive Delayed availability of quality registry data limits timely insight Pre-run analyses are less dynamic and offer less depth than a live dashboard Not all desired analyses can be derived from the dashboard
	**Sharing and validating data in advance**	Sharing data beforehand allows participants to review and validate it and to prepare questions Sharing data in advance removes discussion about patient characteristics, creating room for best-practice exchange	—
	**Workforce organization**	A clear distribution of tasks across complementary roles (content expert, data analyst, project lead) prevents overburdening of individual organizers	Capacity constraints: insufficient personnel to organize the desired number of sessions
	**Funding**	External parties recognize the value of the sessions and provide funding; cost-free facilities are available	—
	**Topic selection and clinical expertise**	Selecting topics based on practice variation Involving clinicians early to ensure clinically relevant topic selection	—
	**Ownership**	Collaboration with professional societies strengthens the session	Balancing the involvement of external stakeholders with hospital autonomy can be challenging
**Setting and format**	**Session format**	Online format lowers the threshold, increases reach and works well for large groups	Online format hampers interaction Online format makes moderation more difficult
	**Group size**	A smaller group supports an efficient session and discussion	Very large groups make discussion difficult to achieve, particularly online
	**Participant composition**	High and consistent attendance and participant variation broaden support; rotation embeds quality improvement within the specialty group A patient representative contributes to useful insights and transparency A multidisciplinary session can be valuable for addressing the full care pathway, ensuring all relevant disciplines are represented Accreditation points attract participants	Logistically difficult to achieve full representation of centres; insufficient or unrepresentative attendance The presence of a patient representative may be daunting for hospitals A multidisciplinary registry requires a tailored approach; diverging topics may hamper interaction
	**Topic focus**	A limited number of topics increases relevance and feasibility Identifying best practices beforehand	Too many or overly broad topics reduce perceived relevance and hamper interaction
	**Tools**	Mentimeter/polls stimulate discussion, capture improvement actions, and promote equal participation Panel discussions and surgical videos stimulate interaction and depth	—
	**Scalability**	A separate session per region is an option for geographically dispersed care A standardized approach and fixed preparatory meetings support scalability and time efficiency	Scalability is difficult when care is dispersed across many centres/regions
**Learning climate**	**Moderation**	A moderator with authority and familiarity within the group Activating discussion through targeted, centre-specific questions	A moderator lacking authority A dominant participant/unequal participation
	**Psychological safety and engagement**	A good atmosphere and safe climate increase interaction, enthusiasm, and engagement Mutual familiarity fosters an open, safe atmosphere	Limited participant input Transparency is difficult to achieve with many centres
	**Dialogue and reflection**	The session stimulates discussion and leads to constructive feedback and knowledge exchange A fixed structure keeps participants engaged	Overly formalizing or documenting improvement points may feel prescriptive and conflict with intrinsic motivation
	**Acceptance of data quality**	Opening the session with an explanation of data quality/analyses prevents unnecessary discussion and frames the aim (improvement rather than explaining data differences) Participants are open to transparent data sharing and accept the outcomes	Data quality discussions may divert attention from the primary aim of the session
	**Organizer motivation**	Intrinsic motivation, a sense of contribution, and inspiration from successful sessions elsewhere	A lack of visible/concrete improvement is demotivating
**Implementation**	**Role of the organizer**	A facilitating, non-directive approach fosters centre engagement and ownership of improvement Active follow-up by the organizer on action points	Lack of a clearly defined role for the registry organization in supporting implementation at the hospital level
	**Role of clinicians**	—	Time constraints among specialists may halt implementation Reliance on a single responsible clinician creates vulnerability
	**Hospital organization**	A designated quality officer provides continuity, available time, and a point of contact for implementation	Differences in organization between hospitals hinder implementation
	**Dissemination of outcomes**	Sharing outcomes and best practices with a wider network (regions, professional society, stakeholders) Success stories motivate other centres	—
**Cyclical learning**	**Session evaluation**	Brief evaluation/feedback round immediately after the session provides valuable input for subsequent sessions	—
	**Follow-up method**	A standardized follow-up procedure (e.g. at 6 months) with structured capture of improvement actions, providing the registry with insight into actual implementation Intrinsically motivated participants initiating follow-up themselves	Centres’ attention to improvement actions fades without active follow-up Logistically challenging to reconvene the same participants The absence of a proven best practice for follow-up Without structured follow-up, the registry lacks insight into whether improvement actions were implemented
	**Session frequency**	A regular cycle (e.g. twice a year) creates continuity and the opportunity to reflect on previous sessions	The ideal frequency does not match the available capacity Limited added value when a topic is repeated without new data being available
	**Recurring topics**	Reviewing themes over time with annual trend analyses Identifying and presenting best practices	The absence of a clear best practice or of recurring topics limits insight into implementation

#### Preparation

The use of interactive data tools was debated: ‘We presented the analyses in PowerPoint—pre-made slides. But participants asked questions we couldn’t answer […]. With an interactive dashboard, you could respond on the spot’ (Organizer 3). Another organizer considered the dashboard as a method to decrease preparation time. Their usefulness, however, may depend on the dashboard’s flexibility: ‘Our available dashboard has a fixed patient inclusion filter […], and lacks the flexibility needed for some sessions; we therefore prefer to run our own analyses, using standardized figures and tables based on dichotomous or median outcomes’ (Organizer 4).

#### Setting and format

During the interview, online sessions were compared with face-to-face sessions: ‘In online sessions, you get almost no response in the chat, and when you ask someone to turn on their camera and say something, they barely do. The polls help make it interactive, and we use a panel discussion to generate the actual discussion’ (Organizer 2). This illustrates that the online format appeared to hamper interaction, although this participant also noted that it seemed to lower the threshold for participation and enable attendance of larger groups. Regarding the composition of participants, an organizer suggested that the attendance of a patient representative could add value: ‘I think the patient representative can provide useful insights’ (Organizer 5).

#### Learning climate

Sharing data in advance was described as a method to prevent debate about data quality: ‘You don’t want the discussion to be about data quality, so I circulate the data beforehand, really asking: is this correct, does anything look odd? But if criticism of certain analyses comes up anyway during the meeting, that can be difficult’ (Organizer 3). Organizers highlighted the relevance of a skilled moderator: ‘We appointed someone with authority […] so that when a discussion moved off into a completely different direction, that person could intervene’ (Organizer 3). Several participants emphasized that group familiarity improved sessions, e.g.: ‘It is a very closely connected group. That, I think, is something exceptional, and it is also why this works well’ (Organizer 5).

#### Implementation

The question of the extent to which the organizer is responsible for implementation and follow-up was raised: ‘It is largely the centres’ own initiative. […] We are not going to oblige a centre to implement certain changes […]. We mainly get the discussion going and provide the facilities […]. I do find that follow-up is a difficult part of this’ (Organizer 4). Reliance on a single clinician was noted as a barrier to durable implementation: ‘If you only have a medical specialist, they are always very enthusiastic, but they all do it on top of their other work. […] If someone falls ill […] it simply stops. It becomes a single point of failure […]. You really need people in the hospital who are permanently employed for this’ (Organizer 5).

#### Cyclical learning

Lastly, one organizer mentioned that recurring topics enable monitoring of improvement over time: ‘It was originally set up to discuss *[a specific complication rate]*, and that now returns every year. Each year it is briefly discussed again: have you changed anything over the past year?’ (Organizer 4).

### Perceived effects of BPMs

Beyond facilitators and barriers, organizers reflected on the perceived effects of BPMs on clinical practice. Organizers reported observing concrete changes in clinical practice following BPMs, including protocol adjustments and adoption of practices from other centres: ‘We are seeing several concrete results. […] We see that meaningful things are being picked up from BPMs that we can actually measure. People said they changed their protocols, or we see the percentage of certain outcomes declining’ (Organizer 1). However, respondents consistently noted that a causal relationship has not been established, as observed changes may reflect other factors, and at least 2 years of follow-up data were considered necessary to detect effects in the registry. Impact was also difficult to assess because variation was not necessarily unwarranted: ‘We sometimes show practice variation, e.g. in dose reductions. But that doesn’t mean that if your hospital is on one end of the spectrum, you’re necessarily doing it wrong. It should be an impulse to look into it’ (Organizer 2). Despite these limitations, organizers regarded the sessions as clinically meaningful and valued them for stimulating discussion about variation. For some sessions, however, the yield remained unclear, and time constraints, loss of focus, or poorly chosen topics could prevent meaningful improvements.

## Discussion

### Statement of principal findings

This study provides the first structured description of BPMs organized across 14 of the 26 Dutch national clinical registries between 2018 and 2025. In total, 55 meetings were held. Most BPMs were organized on a national level and were multidisciplinary. The structured formulation of improvement actions and follow-up was integrated in a subset of the registries. Among BPMs for which evaluations were available, participant satisfaction was generally favourable. Thematic analysis of the interviews identified five themes related to the organization and conduct of BPMs: preparation, setting and format, learning climate, implementation, and cyclical learning. Facilitators included data sharing in advance, skilled moderation, and a safe atmosphere, whereas barriers included online formats, data-quality concerns, and discussing too many topics.

### Strengths and limitations

To our knowledge, this is the first study to comprehensively describe BPMs as a quality improvement instrument in LHCS. Data from BPMs across multiple nationwide registries are presented, and the structural characteristics, facilitators, and barriers to the organization and conduct of BPMs can inform other quality improvement institutes and national registries. Nevertheless, some limitations should be acknowledged. First, the qualitative component was based on interviews with five BPM organizers from a single institution, which may limit generalizability. Moreover, organizers may hold more favourable views toward BPMs, which potentially introduces bias. In addition, not all BPM-level data were available for every meeting. This was most notable for participant satisfaction, reported for only nine BPMs, as structured feedback collection was introduced recently. Therefore, these findings may not be representative of all BPMs. Finally, several facilitators (e.g. sharing data beforehand, limiting topics) were implemented in more recent BPMs. As a result, later BPMs differed from earlier meetings, which may have influenced participant satisfaction scores.

### Interpretation within the context of the wider literature

Evidence on structured, data-driven inter-hospital meetings is scarce and difficult to identify and compare, partly due to the lack of a consistent definition or terminology for these initiatives. In the current literature, similar meetings are described regionally, nationally, or internationally, and as mono- or multidisciplinary [13]. A qualitative study on peer discussions with audit data in Dutch general practice concluded that creating a safe learning environment and patient involvement were key factors to stimulate behaviour change [19]. Although the setting differs from inter-hospital meetings, our participants similarly emphasized the importance of an open and safe atmosphere, as well as the engagement of patients. Patient representatives were involved in a minority of BPMs, and organizers reported they added an in-depth perspective, but also mentioned that their presence may be daunting to HCPs. The optimal role of patient representatives, while preserving the safe environment in BPMs, warrants further investigation. A national quality improvement initiative described in Canada showed similar enablers: trustworthy data and collaboration between HCPs and data analysts [20]. Likewise, this initiative faced challenges related to limited funding and the absence of automated data collection, mirroring the capacity and data-related barriers identified in our study. The similarities with international initiatives suggest that the barriers and facilitators identified in our study are not unique to the Dutch context.

A facilitator identified in our study was the use of recurring themes, which enables comparison of outcomes over time. This aligns with the iterative nature of the PDCA cycle, which requires repeated evaluation to enable continuous quality improvement [9]. However, a recent scoping review on BPMs underscores the challenge of quantifying their impact on clinical outcomes [13]. This was also reflected in our interviews: although organizers observed concrete changes following BPMs, such as protocol adjustments, they noted that quantifying the effects on clinical outcomes remains difficult. They emphasized that effects may be influenced by other factors and require at least 2 years of follow-up before becoming detectable in registry data [21].

### Implications for policy, practice, and research

BPMs appear to provide a practical tool to address inter-hospital variation and support data-driven quality improvement. Rather than demonstrating causal effects on clinical outcomes, this study describes how BPMs operationalize key elements of LHCS by strengthening PDCA cycles. BPMs provide a structured medium to translate benchmarking data into collective reflection, through shared learning across institutions, and formulation of improvement actions. National registries and professional societies may benefit from incorporating BPM-like structures into existing governance frameworks. Our interviews focused on BPM organizers, who provide a broad organizational perspective. Future research could complement this with the experiences of participating health care providers across different specialties and BPMs. Moreover, optimal meeting design, implementation success, and, ideally, effects on clinical outcomes using longitudinal registry analyses should be assessed.

## Conclusion

BPMs are a feasible and well-accepted instrument for facilitating data-driven reflection, shared learning, and action planning across hospitals, and may strengthen LHCS by operationalizing the *check* phase of the PDCA cycle. Identified facilitators and barriers to the organization and conduct of BPMs could guide other comparable quality improvement meetings. Future research should focus on the experiences of health care professionals with BPMs more comprehensively and evaluate implementation success and potential effects on clinical outcomes.

## Supplementary Material

mzag111_Supplementary_Data

## Data Availability

The data underlying this article cannot be shared publicly due to privacy of individuals that participated in the study. The data will be shared on reasonable request to the corresponding author.
