# Supplementary material for: Advancing learning health care systems with best practice meetings: a mixed-methods study with insights from the Dutch Institute for Clinical Auditing
Source: Int J Qual Health Care. 2026 Aug 8;38(3):mzag111. doi: 10.1093/intqhc/mzag111 (PMC13524435; doi:10.1093/intqhc/mzag111)
Supplement: Supplementary file 1 [file mzag111_supplementary_data.zip › Supp.pdf]

Supplementary material

Supplementary File 1. Standards for Reporting Qualitative Research (SRQR) checklist.

Text Section and Item name	Section or Item Description	Page and lines
**Title and abstract**		
**1. Title**	Concise description of the nature and topic of the study Identifying the study as qualitative or indicating the approach (e.g., ethnography, grounded theory) or data collection methods (e.g., interview, focus group) is recommended	Page 1, lines 1-2.
**2. Abstract**	Summary of key elements of the study using the abstract format of the intended publication; typically includes background, purpose, methods, results, and conclusions.	Page 1-2, lines 3-34.
**Introduction**		
**3. Problem formulation**	Description and significance of the problem/phenomenon studied; review of relevant theory and empirical work; problem statement.	Page 3, lines 48-66.
**4. Purpose or research question.**	Purpose of the study and specific objectives or questions	Page 3, lines 67-71.
**Methods**		
**5. Qualitative approach and research paradigm**	Qualitative approach (e.g., ethnography, grounded theory, case study, phenomenology, narrative research) and guiding theory if appropriate; identifying the research paradigm (e.g., postpositivist, constructivist/ interpretivist) is also recommended; rationale**	Page 4, lines 74-75.
**6. Researcher characteristics and reflexivity**	Researchers' characteristics that may influence the research, including personal attributes, qualifications/experience, relationship with participants, assumptions, and/or presuppositions; potential or actual interaction between researchers' characteristics and the research questions, approach, methods, results, and/or transferability.	Page 5, lines 115.
**7. Context**	Setting/site and salient contextual factors; rationale**	Page 4, lines 78-91.

**8. Sampling strategy**	How and why research participants, documents, or events were selected; criteria for deciding when no further sampling was necessary (e.g., sampling saturation); rationale**	Page 4-5, lines 94-100.
**9. Ethical issues pertaining to human subjects**	Documentation of approval by an appropriate ethics review board and participant consent, or explanation for lack thereof; other confidentiality and data security issues.	Page 6, lines 133-137.
**10. Data collection methods**	Types of data collected; details of data collection procedures including (as appropriate) start and stop dates of data collection and analysis, iterative process, triangulation of sources/methods, and modification of procedures in response to evolving study findings; rationale**	Page 4-5, lines 94-98.
**11. Data collection instruments and technologies**	Description of instruments (e.g., interview guides, questionnaires) and devices (e.g., audio recorders) used for data collection, if/how the instrument(s) changed over the course of the study	Page 6, lines 103-118.
**12. Units of study**	Number and relevant characteristics of participants, documents, or events included in the study; level of participation (could be reported in results).	Page 7, lines 143-145
**13. Data processing**	Methods for processing data prior to and during analysis, including transcription, data entry, data management and security, verification of data integrity, data coding, and anonymization/de-identification of excerpts	Page 5 lines 115-116.
**14. Data analysis**	Process by which inferences, themes, etc., were identified and developed, including the researchers involved in data analysis; usually references a specific paradigm or approach; rationale**	Page 6, lines 123-131.
**15. Techniques to enhance trustworthiness**	Techniques to enhance trustworthiness and credibility of data analysis (e.g., member checking, audit trail, triangulation); rationale**	Investigator triangulation: a subset of transcripts was double-coded independently and codes were discussed

		to reach agreement; themes were refined within the wider research team. Page 6, lines 123-138.
**Results**		
**16. Synthesis and interpretation**	Main findings (e.g., interpretations, inferences, and themes); might include development of a theory or model, or integration with prior research or theory	Page 7-10, lines 143-228.
**17. Links to empirical data**	Evidence (e.g., quotes, field notes, text excerpts, photographs) to substantiate analytic findings	Themes and subthemes, with supporting facilitators and barriers, are presented in Table 2; illustrative participant quotes are provided in the Results.
**Discussion**		
**18. Integration with prior work, implications, transferability, and contribution(s) to the field**	Short summary of main findings; explanation of how findings and conclusions connect to, support, elaborate on, or challenge conclusions of earlier scholarship; discussion of scope of application/generalizability; identification of unique contribution(s) to scholarship in a discipline or field	Page 11, lines 238-247.
**19. Limitations**	Trustworthiness and limitations of findings	Page 10, lines 252-260.
**Other information**		

**20. Conflicts of interest**	Potential sources of influence or perceived influence on study conduct and conclusions; how these were managed	End-matter.
**21. Funding**	Sources of funding that supported this work. Role, if any, of the funding organization in the design, implementation, interpretation, and reporting	End-matter.

For Review Only

Supplementary File 2. Table with an overview of the Dutch Institute for Clinical Auditing (DICA) registries that organised BPMs.

**Audit**	**Official registry Name**	**Abbreviation**
Breast cancer	NABON Breast Cancer Audit	NBCA
Colorectal cancer	Dutch ColoRectal Cancer Audit	DCRA
GI endoscopy	Dutch Gastrointestinal Endoscopy Audit	DGEA
Endoscopy complications	Dutch Registry of Complications in Endoscopy	DRCE
Gynaeco-oncology	Dutch Gynaecological Oncology Audit	DGOA
Head and neck oncology	Dutch Head and Neck Audit	DHNA
Hepato-biliary oncology	Dutch Hepato-Biliary Audit	DHBA
Hip fracture	Dutch Hip Fracture Audit	DHFA
Lung cancer	Dutch Lung Cancer Audit	DLCA
Bariatric surgery	Dutch Audit for Treatment of Obesity	DATO
Paediatric surgery	Dutch Paediatric Surgery Audit	EPSA
Prostate cancer	Dutch Prostate Cancer Audit	DPA
Stroke	Dutch Acute Stroke Audit	DASA
Upper GI cancer	Dutch Upper Gastrointestinal Cancer Audit	DUCA
Medication	Dutch Medication Audit	DMA

Abbreviations: best practice meeting: BPM; gastrointestinal: GI; Dutch National Breast Cancer Consultation: NABON.

For Review Only

Supplementary File 3. Survey on structural characteristics of best practice meetings.

This appendix presents the survey instrument used to collect information on structural characteristics on BPMs organised by the DICA.

1. Which registry do you manage?

   (Open-ended)

2. How many best practice meetings have been organised for this registry?

   (Numeric entry)

   (If 0, proceed to question 13; this will be the end of the survey.)

3. At which level were the best practice meetings organised for this registry?

   (Multiple answers possible)

   ☐ National

   ☐ Regional

   ☐ Unknown

4. Were the best practice meetings for this registry mono- or multidisciplinary?

   (Multiple answers possible)

   ☐ Monodisciplinary

   ☐ Multidisciplinary

   ☐ Unknown

5. What was the format of the best practice meetings?

   (Multiple answers possible)

   ☐ Digital (online)

   ☐ In-person (physical)

   ☐ Unknown

For Review Only

6. Was the participation rate (number of attending centres / number of invited centres) recorded?

(Multiple answers possible)

☐ Yes → *If yes, please answer the next question*

☐ No

☐ Unknown

7. What was the participation rate and/or number of participants?

Please specify per best practice meeting if multiple meetings were held.

(Open-ended)

8. Which topics were discussed during the best practice meeting(s)?

(e.g. specific complications, effectiveness of a specific treatment, waiting times, length of stay, etc.)

(Open-ended)

9. Were evaluation tools used to evaluate the BPMs for this registry?

(Multiple answers possible)

☐ Survey

☐ Mentimeter

☐ No

☐ Unknown

10. In how many BPMs were improvement actions and/or best practices formulated during the session?

(Open-ended)

11. Was a (online or in-person) follow-up meeting planned to evaluate the improvement actions?

(Multiple answers possible)

☐ Yes

For Review Only

☐ No

☐ Unknown

Time point 4 – Patient involvement

12. Was a patient representative involved in the preparation of and/or during the best practice meeting?

☐ Yes

☐ No

☐ Unknown

For Review Only

Supplementary File 4. Standard best practice meeting survey.

This appendix presents the standardised survey instrument used to evaluate BPMs organised by the DICA. The survey is administered routinely as part of the BPM program and captures participants' perceptions, experiences, and implementation of improvement initiatives at four predefined time points. All closed-ended items were scored on a 0–5 numeric rating scale.

Time point 1 – Before the meeting

For Review Only

1. On behalf of which centre are you completing this survey?

   (Open-ended)

2. What is your role within this centre?

   ☐ Medical specialist

   ☐ Nurse specialist

   ☐ Other, namely: __________ *(Open-ended)*

3. How often does your specialty group or tumour working group use the Codman Dashboard to gain insight into quality information?

   ☐ Never

   ☐ Annually

   ☐ Quarterly

   ☐ Monthly or more frequently

   ☐ Unknown

4. Have you or someone from your specialty group participated in a DICA best practice meeting in the past two years?

   ☐ Yes

   ☐ No

5. Following participation in this/these best practice meeting(s), were improvement initiatives initiated in your centre?

   ☐ Yes

   ☐ No

6. If yes, which improvement initiatives were initiated?

   (Open-ended)

7. How much confidence do you have in the quality of the data from your own hospital within the quality registry <name of registry>?

   (0 = no confidence at all, 5 = complete confidence)

8. To what extent is there trust within the specialty <name of specialty> to openly discuss (differences in) quality information with colleagues from different hospitals?

   (0 = no trust at all, 5 = complete trust)

Time point 2 – During the meeting

1. Which improvement initiatives did you identify during this part of the meeting?

   (Open-ended)

Time point 3 – Immediately after the first meeting

1. I experienced the best practice meeting as valuable.

   (0 = strongly disagree, 5 = strongly agree)

2. The best practice meeting helped me formulate concrete improvement initiatives.

   (0 = strongly disagree, 5 = strongly agree)

For Review Only

3. I expect that the improvement initiatives resulting from the best practice meeting will be implemented.

   (0 = no confidence at all, 5 = complete confidence)

4. What are the most important success factors for implementing the improvement initiatives formulated during the best practice meeting?

   (Open-ended)

5. What are the most important barriers to implementing the improvement initiatives formulated during the best practice meeting?

   (Open-ended)

6. I felt that there was openness to discuss quality information honestly during the meeting.

   (0 = strongly disagree, 5 = strongly agree)

7. What did you experience as the most valuable aspect of this best practice meeting?

   (Open-ended)

8. What did you experience as the least valuable aspect of this best practice meeting?

   (Open-ended)

For Review Only

Time point 4 – Before the evaluation meeting

1. Our hospital has implemented the improvement initiatives from the best practice meeting.

   (0 = not implemented at all, 5 = fully implemented)

2. What were the main barriers to implementation?

   (Open-ended)

3. What were the main success factors for implementation?

   (Open-ended)

4. What support within your centre would you need in the future to continue implementing improvement actions successfully?

   (Open-ended)

5. What tips or advice do you have for DICA regarding future best practice meetings?

   (Open-ended)

For Review Only

Supplementary File 5. Interview topic guide for BPM organisers.

This appendix presents the topic guide used for the semi-structured interviews with BPM organisers. The interviews were conducted in Dutch; the guide is reproduced here in English translation.

1. Since when have you been involved in best practice meetings, and what has been your role?
2. How many best practice meetings have you been involved in to date?
3. What are your personal experiences with organising best practice meetings?
4. Do you have a standardised approach for organising best practice meetings?
5. Who is typically invited to the best practice meetings?
6. How are participants prepared for the best practice meetings?
7. What methods or tools do you use during the best practice meetings to structure and facilitate discussion?
8. How are the outcomes of the best practice meetings processed and shared with relevant stakeholders?
9. How do you ensure that insights from the best practice meetings actually lead to improvements in care?
10. How do you collect feedback from participants after a best practice meeting?
11. How do you evaluate the success of a best practice meeting? What criteria or measures do you use?
12. What works well during your best practice meetings?
13. What could be improved in your approach to best practice meetings?
14. What are the biggest challenges you have experienced in organising best practice meetings?
15. What have you adjusted or learned from previous best practice meetings?
16. We are developing a protocol for the organisation of best practice meetings. What elements should be included in your view?
17. Do you have a future vision for the further development of your best practice meetings?

For Review Only
